# Exosome based analysis for Space Associated Neuro-Ocular Syndrome and health risks in space exploration

**DOI:** 10.1038/s41526-022-00225-4

**Published:** 2022-09-14

**Authors:** Sudipto K. Chakrabortty, Yevgenia L. Khodor, Robert R. Kitchen, Dulaney L. Miller, Kailey M. Babcock, Kyle S. Manning, Steven P. Lang, Vasisht Tadigotla, Wei Yu, Eric Bershad, Johan Skog, Susana Zanello

**Affiliations:** 1 Exosome Diagnostics, a Bio-Techne brand, Waltham, MA USA; 2 Baylor College of Medicine, Houston, TX USA; 3 KBR, NASA Johnson Space Center, Houston, TX USA

**Keywords:** Biomarkers, Neuroscience

## Abstract

Molecular profiling to characterize the effects of environmental exposures is important from the human health and performance as well as the occupational medicine perspective in space exploration. We have developed a novel exosome-based platform that allows profiling of biological processes in the body from a variety of body fluids. The technology is suitable for diagnostic applications as well as studying the pathophysiology of the Space Associated Neuro-Ocular Syndrome in astronauts and monitoring patients with chronically impaired cerebrospinal fluid drainage or elevated intracranial pressure. In this proof-of-concept, we demonstrate that: (a) exosomes from different biofluids contain a specific population of RNA transcripts; (b) urine collection hardware aboard the ISS is compatible with exosome gene expression technology; (c) cDNA libraries from exosomal RNA can be stored in dry form and at room temperature, representing an interesting option for the creation of longitudinal molecular catalogs that can be stored as a repository for retrospective analysis.

## Introduction

Multiple biological adaptations are provoked by exposure to factors present in space exploration environments. Thanks to many years of sustained human presence on the International Space Station (ISS), the effects of microgravity, radiation, altered inspired gas composition, stress, isolation and other factors have been studied in humans by physiologic observations, blood and urine biochemistry and medical monitoring. In our systems biology era, deep molecular characterization of the individuals’ response to such unique environmental scenarios should also include a complete survey of the longitudinal transcriptomic profiles. We have developed a novel exosome-based approach that allows profiling of biological processes in the body from a variety of body fluids. Exosomes and other extracellular vesicles (EVs) are nanoscale in size (typically 50–200 nm in diameter) and released from all living cells to end up in body fluids such as urine, plasma, or cerebrospinal fluid (CSF). Exosomes contain molecular genetic material from the cell of origin, including the RNA transcriptome, and allows monitoring of tissue-based changes from an easily accessible body fluid. With the use of this platform, we can profile the entire RNA transcriptome from exosomes in urine, plasma or CSF which opens a window into studying specific transcriptome changes in the body, including mapping pathways that are affected in the target population^1^. We are now utilizing this approach to answer questions regarding the fundamental mechanisms underlying one of the current high-focus risks of human spaceflight, the Spaceflight-Associated Neuro-ocular Syndrome (SANS).

SANS comprises ocular structural and visual manifestations associated with the exposure to microgravity, especially during long-duration missions on the ISS^2^. Signs include optic disc edema, optic nerve sheath distension, increased retinal and choroidal thickness, cotton wool spots, and posterior globe flattening with decreased near visual acuity^3,4^. Spaceflight-associated peripapillary optic disc edema and substantial choroid thickening have been observed bilaterally in both sexes^5^. Similar signs have been reproduced in the ground model of simulated microgravity head down tilt bed rest^6^. While it has been hypothesized that elevation of intracranial pressure (ICP) may be associated with SANS, ICP has not yet been thoroughly investigated in astronauts and the question remains to be answered. For some, ICP increase alone may not be responsible for SANS^6^. Genetic determinants of 1C metabolism enzymes have been shown to be associated with SANS^7^. In view of this, we have embarked on a quest to characterize the SANS risk by performing a longitudinal profile at the transcriptomic level in astronauts participating in long-duration missions on the ISS, to discover susceptibilities, risk factors, and uncover underlying processes leading to SANS. This is the objective of a NASA-approved flight study in astronauts for which subject recruitment has just started^8^.

### This article reports some technical proof-of-concepts in preparation for such study

In a pilot phase, we previously documented that a terrestrial analog of increased ICP is characterized by inflammatory transcriptional signatures in the CSF of affected individuals through the analysis of exosomes, a subtype of EVs, that are derived from living cells and present in biofluids^9^. While an imperfect analog^7^, idiopathic intracranial hypertension^10^ (IIH) exhibits some of the characteristics of SANS and it is conceivable that certain pathophysiology may be shared between the two conditions. In this paper, we expand on those findings by further investigating different biofluids in IIH patients undergoing CSF drainage as a therapeutic intervention to decrease ICP. We utilized a newly developed RNA-Seq workflow and analysis pipeline that gives a much broader view of the exosome content than what has been typically published on exosomes. Most exosome RNA studies in the literature focus on small RNAs (such as miRNAs) because of the electropherogram size distributions, however, here we show a much broader diversity of detected long RNAs, and achieved a similar diversity of RNAs as to when sequencing a tissue sample^11,12^. In addition, we tested the currently used in-flight urine collection tubes to assess their compatibility with this whole transcriptome profiling technology. Finally, we further explored the potential ease of use for the exosome technology through the preservation of exosomal genetic material in dry form to create a molecular catalog of an individual, that can be further applied as a standardized tool in the space medical and research kit of upcoming exploration scenarios.

## Results

### RNA content and quality

**Fig. 1:**
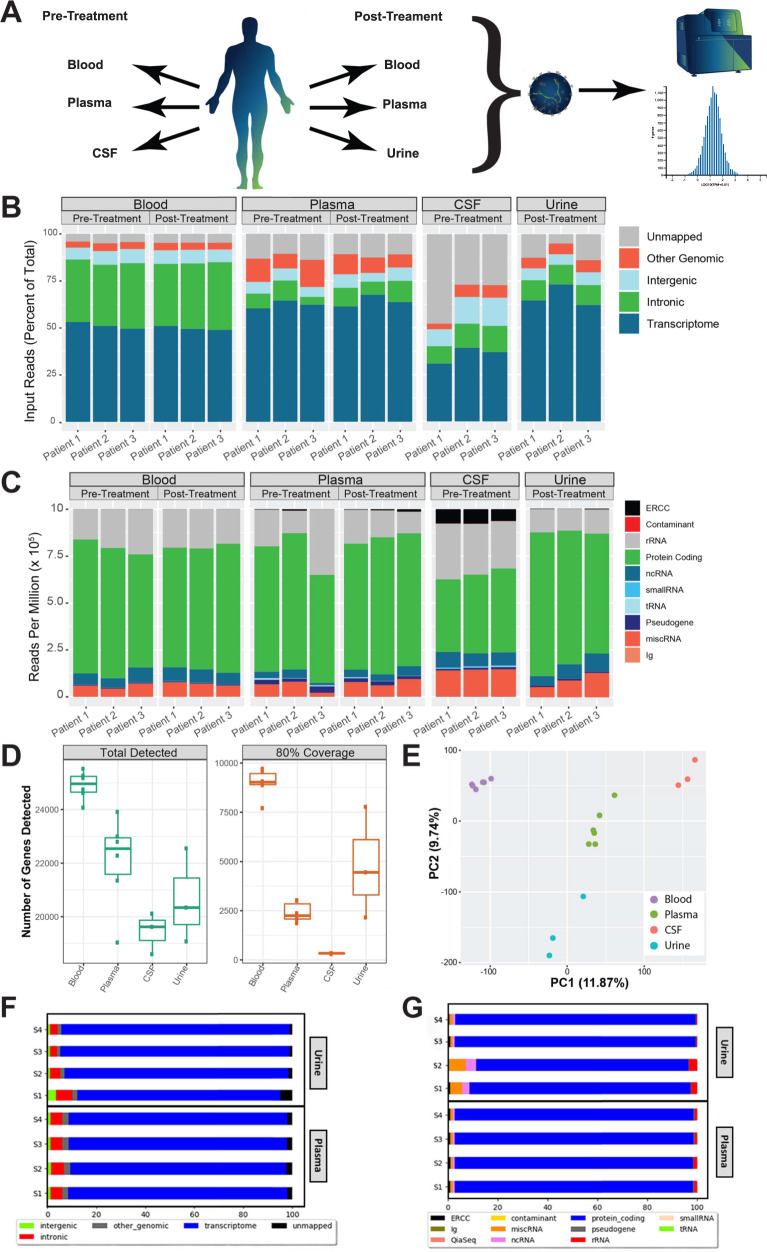
High quality total RNA-Seq data on long RNAs extracted from extracellular vesicles from four different biofluids. **A** A schematic summarizing experimental procedure. Whole blood, plasma, and CSF were collected from patients exhibiting high intercranial pressure (ICP) at their initial visit (“Pre-Treatment”) and whole blood, plasma, and urine at a follow-up visit (“Post-Treatment”) were subject to exoRNA isolation, RNA-Seq, and data analysis. **B** Stacked bar graphs showing the percentage of RNA-Seq reads mapping to transcriptome, intronic, intergenic, and other genomic regions by sample and biofluid type. **C** Stacked bar graphs showing the number of reads per million (RPM) mapping to various biotypes by sample and biofluid type. **D** Boxplots of the number of total genes detected and the number of genes covered at 80% or higher by biofluid. The center line represents the median, boxes represent ±1 interquartile range, and whiskers ±1.5 interquartile range. **E** Principal component analysis (PCA) of all samples showing clear separation of samples by biofluid type. **F** Exome capture performed on both plasma and urine RNA-Seq libraries resulted in increased proportion of reads mapped to the transcriptome when compared to total-RNA seq. **G** Exome capture performed on both plasma and urine RNA-Seq libraries resulted in increased proportion of reads mapping to protein coding genes and depletion of reads mapping to rRNA when compared with total RNA-Seq.

Our first objective was to investigate and compare the nature of the exosome RNA population in the various types of biofluids. For this purpose, we collected whole blood and plasma before and after lumbar puncture (LP) in IIH patients reporting to the neurology clinic. Patients presented with an elevated ICP (before LP) which was reduced after CSF drainage by LP. Patients then returned to the clinic after 1 month to provide a second blood sample. CSF was collected at the time of the first visit, and urine was collected during the follow-up visit. We isolated EVs from plasma, urine, and CSF prior to RNA extraction using optimized methods from Exosome Diagnostics (Waltham, MA, USA). We also isolated intracellular RNA from whole blood stabilized in PAXgene^®^ Blood RNA Tubes (QIAGEN, Hilden, Germany) as a comparison. Total RNA-Seq libraries were made from isolated RNA, and gene expression analysis was performed (Fig. 1A). Hybrid capture-based enrichment of the RNA exome significantly improves the proportion of reads mapping to the transcriptome and protein-coding genes (Fig. 1F, G). However, since the entire exosomal RNA transcriptome (including long non-coding RNAs) were being investigated in this study, results are shown for whole transcriptome data rather than exome-capture.

The quality of sequencing from EV RNA in different biofluids was of primary interest. While the proportion of mappable reads differs between biofluids, we observed that overall, the majority of the reads mapped to the transcriptome (Fig. 1B). Notably, samples collected from whole blood had a higher proportion of reads that map to introns. That finding is consistent with randomly primed RNA-Seq libraries from the whole blood sample containing nucleic acids from cells that include introns and pre-mRNAs that have not been completely processed into mature transcripts^13^. Human introns are much longer than coding regions and will account for a disproportionate number of reads, lowering the quantity of reads that hit informative regions of the transcriptome in each sample. Of the EV RNA samples, CSF samples contained the fewest mappable reads, most likely due to the fragmented nature of the recovered RNA. We did not try to map sequences shorter than 30 nts (Supplementary Fig. S1). Ribosomal RNAs were depleted from each sample since they would otherwise consume most of the reads. We observe that most transcriptome-mapping reads in all biofluids map to protein coding genes (Fig. 1C). CSF samples had relatively fewer reads mapping to protein coding genes, most likely due to a higher proportion of fragmented RNAs in these samples resulting in less efficient ribodepletion. In addition to protein coding genes, we also detected ample non-coding RNAs and small RNAs. ERCC synthetic RNA spike-ins were added to each sample as a spike-in quality control. This demonstrated a greater than 0.9 correlation coefficient (Pearson’s) between samples, underscoring excellent technical reproducibility of the RNA-Seq workflow. It also showed a large dynamic range of detection of five orders of magnitude in each sample (Supplementary Fig. S2). The relative proportion of ERCC spike-ins was greatest in CSF due to the lower exosome RNA yield from those samples. Whole blood allows for detection and coverage of the greatest number of genes, as expected from a sample that contains peripheral blood mononuclear cells and red blood cell RNA (Fig. 1D). Interrogating plasma and urine EV RNA allowed for detection of over 20,000 genes on average at this sequencing depth. We also looked at the number of genes that were detected with at least 80% gene body (exons only) coverage. This metric revealed a very high quality of the urine EV RNA, where 4448 genes were detected with at least 80% coverage (Fig. 1D). The corresponding number for plasma and CSF was 2245 and 334, respectively. Notably, the gene expression in each biofluid is different enough that each clearly segregates with its replicates in a principal component analysis (PCA) (Fig. 1E).

### Biomarker discovery for IIH

**Fig. 2:**
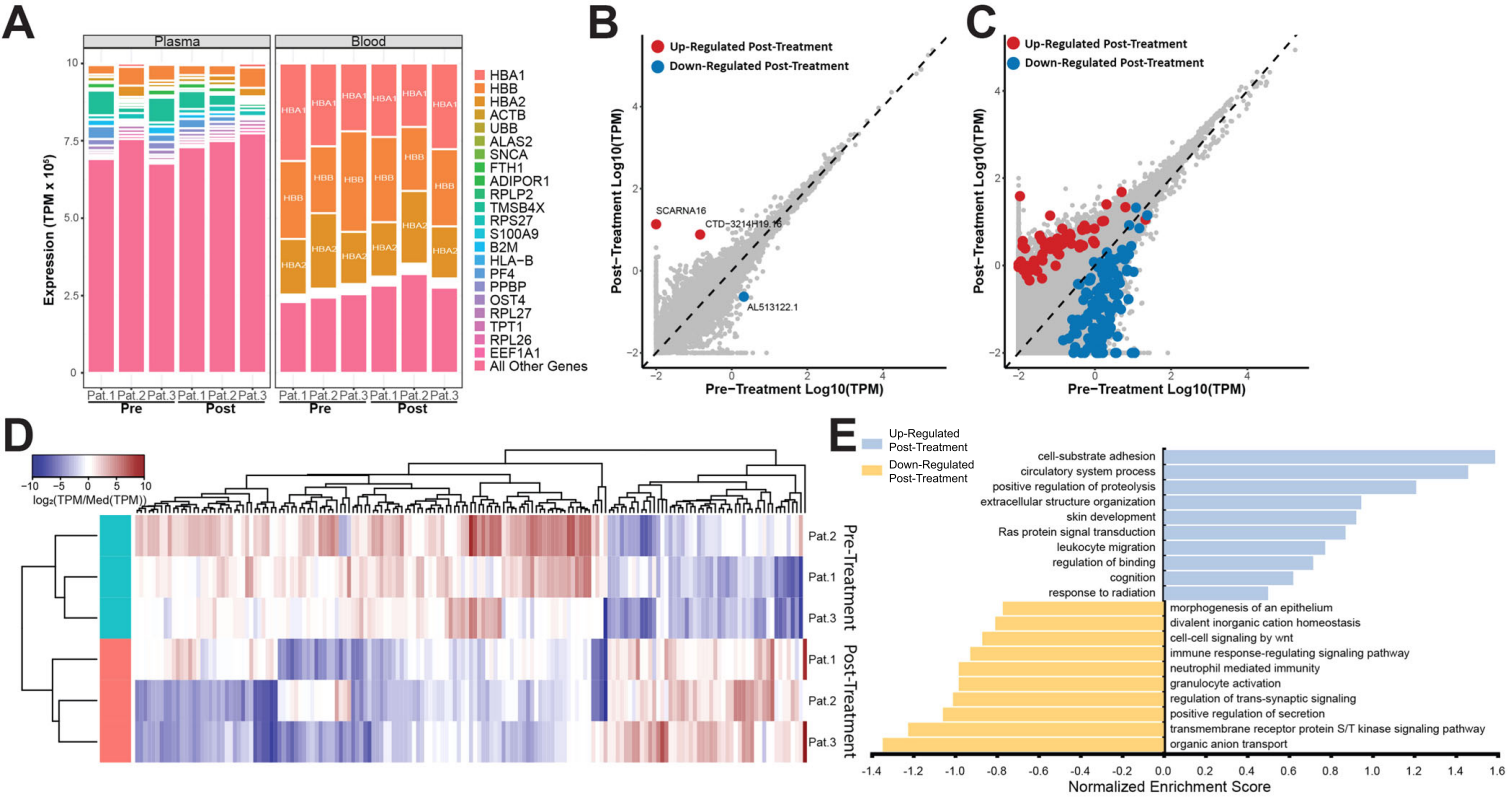
Differential gene expression analysis in plasma is more informative than in whole blood samples from ICP patients. **A** Stacked bar graphs showing the expression (in TPM) of the ten most abundant detected genes in individual plasma and whole blood samples. Pink bars on the bottom represent abundance of all other genes detected. **B** Scatterplot of all genes detected in pre- and post- treatment whole blood samples. Genes that are significantly up-regulated (p-adj. (Benjamini-Hochberg) < 0.05) in post-treatment samples are shown in red, genes that are significantly down-regulated (p-adj. (Benjamini-Hochberg < 0.05) in post-treatment samples are shown in blue. **C** Scatterplot of all genes detected in pre- and post- treatment plasma samples. Genes that are significantly up-regulated (p-adj. (Benjamini-Hochberg) < 0.05) in post-treatment samples are shown in red, genes that are significantly down-regulated (p-adj. (Benjamini-Hochberg < 0.05) in post-treatment samples are shown in blue. **D** Heatmap showing differential expression of significantly differentially expressed genes (p-adj. (Benjamini-Hochberg) < 0.05) between pre-treatment (teal) and post-treatment (salmon) plasma samples of three high-ICP patients. **E** Gene set enrichment analysis of gene expression in pre- vs. post-treatment high-ICP plasma patient samples (p-adj. (Benjamini-Hochberg) < 0.05).

A primary objective of this analysis was to compare the advantage of using EV RNA isolated from plasma vs. using whole blood for biomarker detection from the same subjects. In order to compare the two biofluids, we examined the diversity of gene expression information in individual samples. The whole blood RNA was extracted from a 10 ml PAXgene^®^ RNA tube (2.5 ml blood + 7.5 ml preservative) and the EV RNA was isolated from 2 ml plasma. We determined the top 10 expressed genes in whole blood and plasma and ranked them by their expression abundances and compared that to the remaining informative transcriptome targets. In blood, three genes, HBA1, HBB, and HBA2, accounted for almost 75% of the informative transcriptome reads in each sample. While these genes were also present in plasma EVs, their share of transcriptome reads was significantly smaller (Fig. 2A) leaving more diversity of RNA targets for biomarker discovery efforts in the EVs. From the biomarker discovery efforts, we could only detect three differentially expressed genes between the pre-treatment and post-treatment whole blood samples (Fig. 2B), while 185 differentially expressed genes were detected in comparable plasma EV samples (Fig. 2C, D). We observed that the majority of differentially expressed genes in plasma were downregulated post-treatment (Fig. 2C), and we were able to discern individual transcriptome differences between patients (Fig. 2D). To gain further insight into the EV RNA changes following treatment for ICP, we performed a gene set enrichment analysis (GSEA) on the significant differentially expressed genes in plasma to see whether these genes could be grouped for further investigation. GSEA allows for statistical determination of whether groups of genes that share biological function are expressed differently between two phenotypes, such as high vs. low ICP. While this analysis was limited to only three patients, we observed that the up-regulated gene sets in post-treatment plasma point toward genes implicated in adhesion, extracellular structures, binding, and migration, while the downregulated genes include immune response signaling and neutrophil mediated immunity (Fig. 2E). A greater sampling of ICP patients may be needed to better understand the significance of these gene sets.

**Fig. 3:**
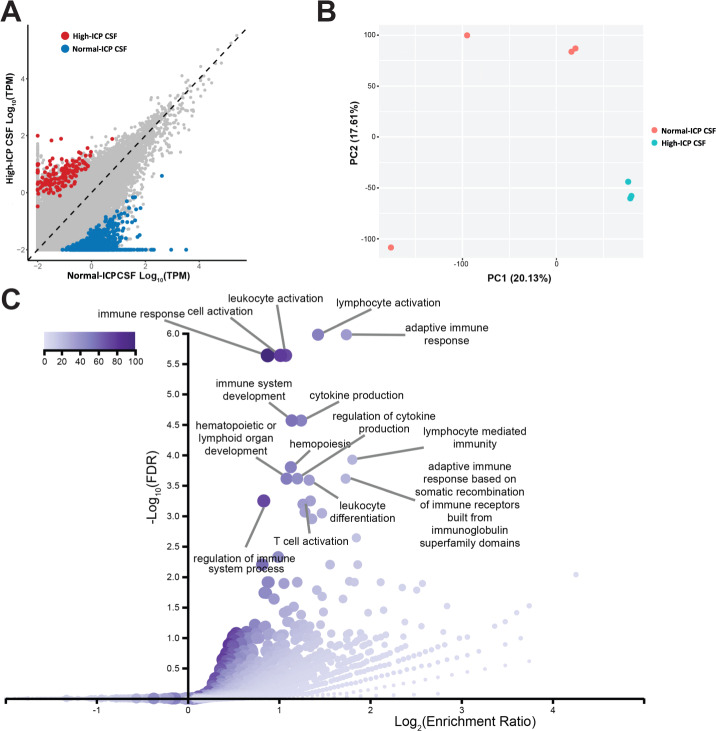
Patients with high ICP have a different gene expression profile in the CSF extracellular vesicles. **A** Scatterplot of all genes detected in high-ICP CSF from patients and normal-ICP CSF samples from a biobank. Genes that are significantly higher (p-adj. (Benjamini-Hochberg) < 0.05) in high-ICP patients are shown in red and genes that are significantly higher in normal-ICP samples (p-adj. (Benjamini-Hochberg) < 0.05) are shown in blue. **B** PCA of high-ICP (represented in teal) and normal-ICP CSF samples (represented in salmon). **C** Gene ontology (GO) analysis for genes that are enriched in normal-ICP CSF samples relative to high-ICP patient CSF showing the top 15 enriched GO terms and their relative significance by depth of color. Enrichment is shown on the *x* axis, and false discovery rate (FDR) on the *y* axis.

Unlike whole blood and plasma samples, CSF samples were only obtained from the LP at the first visit (high-ICP). The goal with the CSF samples were to identify genes that become upregulated due to the high-ICP, so these samples were compared to CSF collected from four pools of CSF from biobanked controls (BioIVT, Westbury, NY, USA) with normal-ICP. In contrast to blood and plasma samples, CSF differential expression analysis revealed large gene expression changes in CSF from patients with high-ICP. We found 777 genes up-regulated and 134 genes down-regulated in normal-ICP samples compared to the high-ICP CSF samples (Fig. 3A). The large number of differentially expressed genes is further exemplified by the large difference on the PCA plot between normal-ICP CSF and high-ICP patient CSF (Fig. 3B). This indicates that the high-ICP drives molecular changes in cells that can be monitored by the exosome RNA transcriptome. To further characterize these differences, we looked at gene ontology (GO) differences between samples. The top 15 biological process categories for genes whose expression is significantly higher in normal-ICP samples are shown in Fig. 3C. These categories are heavily implicated in immune response, indicating the patients with high ICP have a significant decrease in immune associated genes. In contrast, there were no significant biological process categories for the high-ICP high genes. We summarized GSEA results based on MSigDb (see Supplementary Fig. S3).

**Fig. 4:**
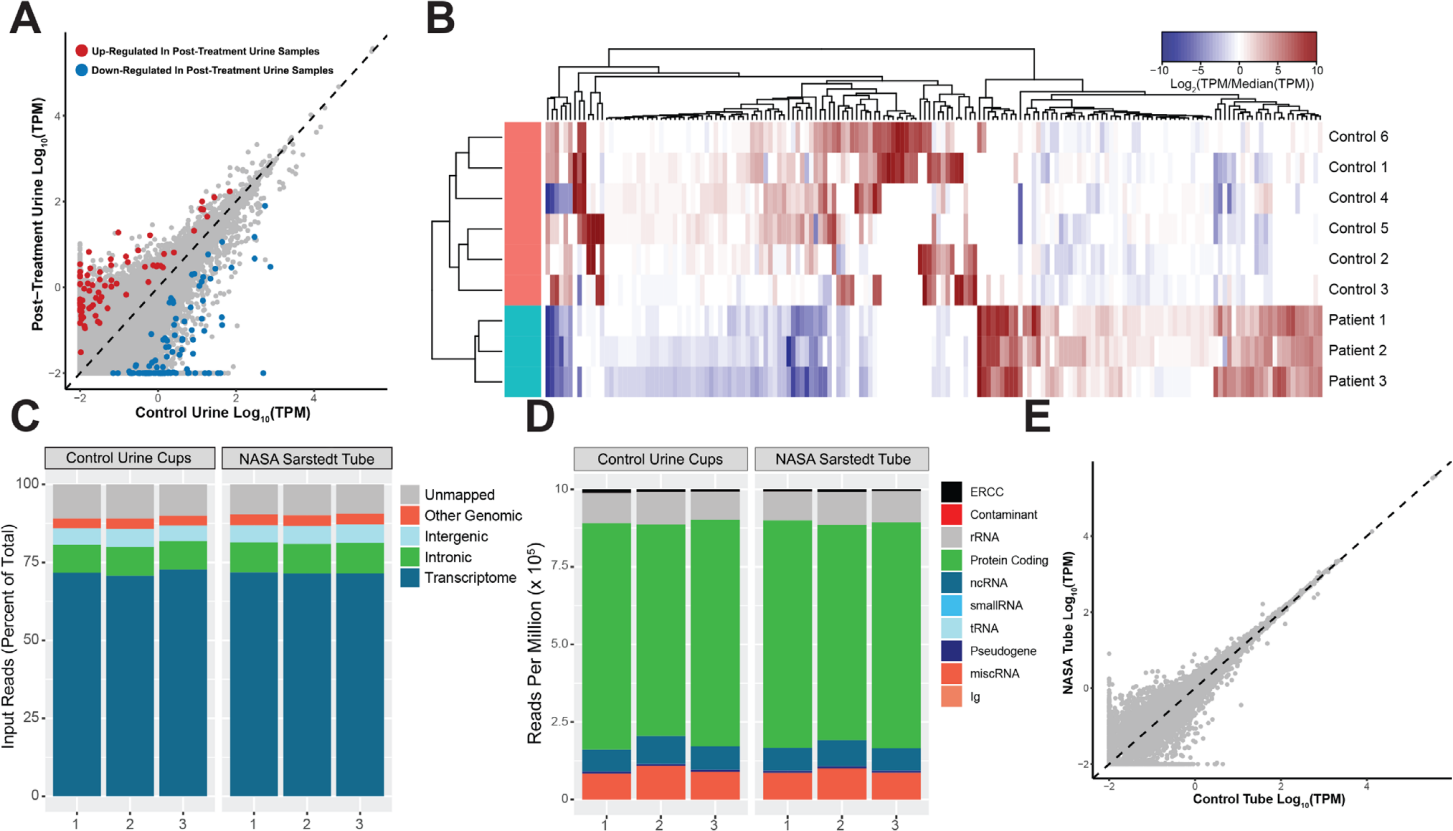
Urine exosome RNA profiles from post-treatment urine samples and feasibility of using the NASA Sarstedt tube for urine collection and exosome RNA readout. **A** Scatterplot of all genes detected in post-treatment patient and control urine samples. Genes that are up-regulated in post-treatment patients are in red and genes that are down-regulated in post-treatment patients are in blue. **B** Heatmap showing differential expression of significantly differentially expressed genes between post-treatment patient (teal) and healthy control (salmon) urine samples. **C** Stacked bar graphs showing the percentage of RNA-Seq reads mapping to transcriptome, intronic, intergenic, and other genomic regions of exoRNA derived from urine collected and stored using Exosome Diagnostics control urine cups and NASA Sarstedt collection tubes. **D** Stacked bar graphs showing the number of reads per million (RPM) mapping to various biotypes of exoRNA derived from urine stored using Exosome Diagnostics control urine cups and NASA Sarstedt collection tubes. **E** Scatterplot of all genes expressed in urine samples stored using Exosome Diagnostics control urine cups and NASA Sarstedt collection tubes.

In contrast to the CSF samples, urine samples from high-ICP patients were only collected at a follow-up (second) visit, so we do not have a sample that coincides with the diagnosis of high ICP. Nevertheless, we compared the collected post-treatment urine samples to six control urine samples from a biobank. Differential expression (DEx) analysis found only 120 genes upregulated and 89 genes down-regulated in the control urine samples (Fig. 4A, B). GO analysis on these genes did not result in any statistically significant categories (data not shown) and PCA analysis did not segregate the post-treatment samples from the control urine samples (Supplementary Fig. S4). This result is in alignment with our expectations since the urine samples were collected post-treatment, after the ICP state in the patient was resolved and the patients were not presenting with further symptoms. Future studies using samples collected at time of diagnosis may reveal if changes in urine EV RNA from high-ICP patients can reveal biological insights.

One potential pitfall in the current workflow is that urine collection tubes currently used by NASA were not the same as the standard urine cups used by Exosome Diagnostics during development. Thus, it was vital to determine whether the urine collection using the standard collection methods from ordinary clinics and the Sarstedt^®^ tubes (Sarstedt AG & Company, Nümbrecht, Germany) available for NASA in-flight urine collection produced comparable results. To do so, we collected healthy urine samples and pooled them into one sample. Pooled samples were divided among three standard collections and three Sarstedt^®^ tubes and incubated for 24 h at 4 °C to mimic storage and transport. Following incubation, EVs were captured, and RNA was isolated from them. Long RNA-Seq libraries were prepared as previously described using Exosome Diagnostics’ protocols. As summarized in Fig. 4C, E, we observe no difference in sample quality between the two collection tubes. In both, we observed almost 75% of sequenced reads mapping to the transcriptome (Fig. 4C), ~75% transcriptome reads mapping to protein coding genes (Fig. 4D), and no significant differences in gene expression (Fig. 4E). We therefore conclude that Sarstedt^®^ collection tubes are compatible with current EV isolation methods and can be used for in-flight collection.

Finally, we tested whether cDNA libraries synthesized via this workflow could be preserved in dry form for future use. This test forms part of a wider hypothesis for the development of a universal platform for longitudinal surveillance of astronauts and their health status. The data showed that cDNA libraries can be stored desiccated and at room temperature for at least a month (Supplementary Fig. S5). Longer duration stability will be also assessed to determine the shelf life of the desiccated libraries. We tested the efficacy of three commercially available preservation agents (DNAstable^®^, Whatman^®^ FTA^™^ elute card (Sigma-Aldrich, St. Louis, MO) and GenTegra-DNA^®^ (GenTegra, Pleasanton, CA)) and found that the three preservatives were equally able to yield high quality sequencing data, with sequencing metrics consistent between pre- and post- desiccation libraries (Supplementary Fig. S6). Differences between individual libraries were larger than those differences arising from the preservation method. Only the DNAstable^®^ eluate was compatible with the Bioanalyzer quantification and sizing analysis. In weighing all these characteristics, we have adopted DNAstable^®^ for further use when libraries are to be preserved in dry form.

## Discussion

The significance of the specific profile of the exosomal RNA for each biofluid is that it allows researchers to determine the most appropriate sample to use for addressing a particular question. We observed tissue-specific gene enrichment for each kind of biofluid, based on gene ontologies performed during the gene expression analysis (Supplementary Tables 1, 2). We undertook this examination to ascertain that the samples planned to be collected in the upcoming SANS flight study would yield good quality data. The flight samples are irreplaceable due to the small number of astronauts and the unique longitudinal collection times along the mission. We can now ascertain that plasma EVs and urine EVs will provide the most appropriate sample and gene coverage, with plasma being the most informative from the perspective of all physiologic systems, while urine carries a gene enrichment from the cells within the urinary tract (e.g., kidney, bladder, and prostate if male). This urinary tract gene enrichment is due to the glomerular filtration apparatus, which prevents most blood-borne vesicles from entering the nephron^14–17^. While CSF generally had less RNA, it was found to be enriched for brain and immune system-related genes. These EV RNA sources will be important to answer questions related to the nature of ICP-associated conditions of our interest, namely, IIH and SANS. It has previously been shown that endothelial cells exposed to microgravity change their RNA transcriptome and depress the pro-inflammatory cytokine secretion^18^. We show for the first time that similar transcriptome information can be assessed through exosome RNA analysis from biofluid samples. Exosome technology has only recently begun to be applied to space biology and thus, our state-of-the art methods will prove an innovative application of this emerging field. Examples of investigations of EV content in spaceflight samples are very scarce. Recent retrospective investigations of EV content in archived plasma samples from astronauts before and after short (7–15 days) missions to the ISS have shown that mt‐DNA (MT‐CO1 and MT‐CO3) levels were increased only in plasma as cf‐mtDNA rather than EV‐mtDNA^18^.

Because the methods for processing whole blood and plasma have impacts of logistic importance during spaceflight, we needed to determine which type of sample would yield better data in prospective studies. When deciding on which biofluid to utilize, a few key considerations need to be addressed; the feasibility of collection, the unique exosomal RNA cargo of each biofluid, and the clinical condition for the downstream evaluation. First, via analysis of genes detected from blood, urine, plasma, and CSF, we observed that a large number of exosomal RNAs overlap between biofluids (Supplementary Fig. S7). This exemplifies the unique ability of plasma exosomal RNA in which signals from all over the body are still detectable regardless of anatomical proximity. However, it is still clear that each biofluid provides unique RNA signatures enriched to their respective anatomical features (Supplementary Figs. S7, S8). For example, GSEA of genes upregulated in urine when compared to plasma revealed significant enrichment of urogenital system-related features (Supplementary Fig. S8C). Similarly, GSEA analysis of genes up in CSF when compared to plasma revealed significant enrichment of neuronal and nervous system- related features (Supplementary Fig. S8D). However, due to the more invasive nature of CSF collection, it would be worth the investigation of biomarkers that overlap with CSF that come from plasma and urine to provide adequate information from the astronauts prospectively experiencing microgravity. One way to do this is to enrich neuronal exosomes in plasma or urine via surface proteins present in brain-derived exosomes. This presents another unique capability of exosomes in which it may be possible to enrich for a subpopulation of exosomes based on surface protein of interest.

Since cells from whole blood contains a predominant population of hemoglobin-encoding genes, the representation of all other genes carrying relevant information from the systems are eclipsed by the highly abundant globin genes. In contrast to the whole blood cells, exosomes are released by a variety of cell types, including from the central nervous system, and carry RNA transcriptome information from cells not circulating in blood. In fact, upon performing differential gene expression analysis on samples between high and normal ICP (pre- and post-LP, respectively), we were able to identify a significant number of differentially expressed genes in plasma, but only a few in whole blood. In this proof-of-concept study, we were able to perform gene enrichment analysis pointing to some candidate pathways that may be impacted by high ICP, but these warrant further investigation in a larger scale study. In addition, it would be a major advance for monitoring IIH patients and other patients with chronically impaired CSF drainage (such as normal pressure hydrocephalus, and/or obstructive hydrocephalus) to have a urine or blood test that can diagnose elevated ICP without having to subject patients to repeated radiation from a head CT. These types of EV studies have implications for both astronaut and on-earth syndromes. For example, exosome RNA analysis from urine samples is already used as a diagnostic for prostate cancer (ExoDx^™^ Prostate test). This test has been used clinically since 2016 and is included in the NCCN guidelines for early prostate cancer detection^19,20^.

Exosome RNA from CSF samples of patients presenting with high ICP clustered as distinctively different groups and gene expression analysis and enrichment revealed the involvement of immune processes in agreement with previously reported results on IIH patients^8^.

Also important for prospective flight studies was to test the compatibility of the currently used urine collection tubes with our methods. Our results demonstrate that the routine methods for urine collection in flight can be used for the application of exosome technology. Our results also allow the option of retrospectively investigating previously collected samples using these tubes and which have been stored in NASA repositories along the history of human spaceflight.

Finally, we report on an aspect that constitutes an advancement in exploration capabilities given its promising compatibility with the operational requirements in space environments, as explained below (Supplementary Fig. S5). This is an innovative conception for a health surveillance system consisting of a repository of genomic/transcriptomic information in the form of desiccated amplified DNA/cDNA libraries. Establishment of an astronaut medical library to preserve highly stable pre-processed biofluid samples and associated digital data is intended to provide the evidence base to track hazardous exposure-driven, measurable changes in crew members. Applications can include risk management for today’s low earth orbit astronauts, precision medicine applications for exploration astronauts, in mission health assessments for extraplanetary crews and commercial space travelers, and a serial investigation of the long-term health consequences of spaceflight across various exposures and partial G-loads. In particular, the identification of those applications where actionable measures can be obtained is of high interest.

Medical conditions are likely to develop during space exploration missions, and the likelihood of their occurrence depends on the health risk, specific mission events and circumstances, and individual predisposition. An impactful genomics program within research and clinical operations to complement existing crew health surveillance pipelines is currently an unmet need in space exploration. The documentation of the adaptations and responses to spaceflight informs human health risk assessments, whose mitigation is needed to ensure crew health and performance. This provides the rationale for creating a genomics and transcriptomics repository. Being able to use exosomes from biofluids such as urine and plasma can provide a window into tissues/processes that are otherwise not easily accessible. The fact that we can do complete RNA transcriptome profiling from these biofluids can give important insights into systemic as well as tissue-specific changes in these individuals and enables baseline readings as well as an easily accessible longitudinal monitoring platform.

The innovation of the “medical library” concept resides in the following characteristics:

- A standardized, longitudinal biospecimen repository in the form of genomic/transcriptomic information (amplified DNA/cDNA libraries) would constitute a universal form of storing stable, measurable biologic samples for astronaut health surveillance, with the option of adding converted digital surveillance data from crew members.
- The repository would be ready for use as pre-processed biologic samples, will be durable, and not need any freezer space until required for analysis and digital transformation.

The recovery and usability of desiccated libraries constitutes a process innovation combining tested existing technologies, but never used in this fashion before.

The proof-of-concept demonstrated that DNA libraries were successfully preserved at room temperature over a period of 1 month, documented by a sequencing profile that did not differ from that of the original historic libraries. Longer preservation times are still being tested. This technology also has benefits that extend beyond its applications in space exploration. Incorporating medical libraries for periodic screening of workers in hazardous conditions could become a valuable tool in occupational health. Medical libraries could also be used in clinical trials to monitor the response or lack thereof to new pharmaceutical compounds.

## Methods

### Sample collection

Methods were performed in accordance with relevant regulations and guidelines. The study was reviewed and approved by the institutional review boards at Johnson Space Center and Baylor College of Medicine (BCM), Houston, TX. Subject recruitment was done at BCM and written informed consent was obtained from all subjects.

The study population was a pool of neurological patients requiring therapeutic LP. The subjects had a LP in the lateral decubitus position with legs slightly extended. Once the needle was in the thecal sac at the L3-L4 or L4-L5 region, the manometer was observed for the presence of pulse and respiratory waves to indicate patent communication between needle and subarachnoid space. Next, the opening pressure was monitored over a period of 5 min, ICP was measured every minute, and the average calculated. CSF was then drained as per the normal clinical procedure to reduce ICP. 5 ml was collected specifically for the purpose of this study. Three subjects were recruited for this pilot study.

In addition, subjects had 10 ml blood drawn for plasma separation in K_2_EDTA BD Vacutainer^®^ Blood Collection Tubes with gel barrier, and in PAXgene^®^ tubes for whole blood collection. Blood was collected at the time of the first visit (when the patient had elevated ICP) and on the second visit (when the patient had reduced ICP). CSF was collected only on the first visit. Urine was collected only on the second visit. All plasma, urine and CSF samples were pre-filtered through a 0.8 µm syringe filter prior to EV processing, aliquoted and stored at −80 °C in a freezer at the Center for Space Medicine at BCM and then shipped to the Exosome Diagnostics facilities in Waltham, MA. The PAXgene^®^ Blood RNA Kit was used for isolation and purification of cellular RNA from blood stabilized in PAXgene^®^ Blood RNA Tubes according to manufacturer’s recommendation. Six de-identified normal control urine samples were also processed from individuals with no known ICP pathology.

### EV RNA isolation

Extraction of EVs including exosomes from plasma, CSF, and urine samples was carried out in one central laboratory by Exosome Diagnostics (Waltham MA, USA). 2 ml of plasma, 20 ml of urine, and 3 ml of CSF were used for extractions as previously described^19,21^. In this setting any lipid vesicles smaller than 800 nm in diameter were isolated.

### RNA-Seq

Total RNA-Seq library construction and sequencing were performed at Exosome Diagnostics (Waltham, MA) using the proprietary EV long RNA-Seq platform. Briefly, isolated RNA samples from whole blood, plasma, CSF, and urine were first treated with DNase to remove any trace amounts of co-purified DNA present in the sample. Post-DNA digestion, synthetic RNA spike-in controls (ERCC spike-in mix, Thermo Fisher Scientific) were added to each sample. The exoRNA/ERCC blend was then reverse transcribed using a combination of random hexamers and oligo-dT primers. Second strand synthesis and adapter addition were performed using a PCR-based approach. Libraries were purified via two rounds of AMPureXP^®^ beads (Beckman Coulter), and ribosomal cDNA was depleted enzymatically. The final libraries were then amplified to desired concentration for sequencing and purified using AMPureXP^®^. Libraries were quantified using the Agilent Bioanalyzer^™^ High Sensitivity Assay (Agilent Technologies, Santa Clara, CA, USA) and Qubit 1X dsDNA HS Assay Kit (Thermo Fisher Scientific, Waltham, MA, USA). Libraries were pooled and sequenced on Illumina^®^ NextSeq500 using 2 × 150 cycles read length chemistry.

#### Hybrid capture

Libraries created from 2 ml plasma and 10 ml urine samples were pooled by mass prior to hybridization capture. Libraries were hybridized to a human whole exome plus UTR biotinylated probe set with a 1 h hybridization time. Hybridized DNA was then captured using streptavidin-coated beads at 70 °C through a series of wash buffers. PCR amplification was performed to generate enough material for sequencing and then purified using a single round of AMPureXP^®^ beads (Beckman Coulter). Libraries were quantified using the Agilent Bioanalyzer^™^ High Sensitivity Assay kit (Agilent Technologies, Santa Clara, CA, USA) and Qubit 1X dsDNA HS Assay Kit (Thermo Fisher Scientific, Waltham, MA, USA). The final capture was diluted to a desired concentration and sequenced on Illumina^®^ NextSeq550 using 2 × 150 cycles read length chemistry.

### Bioinformatics

2 × 150 paired-end reads were aligned to the human genome (hg38) with the Gencode v25 gene model using STAR (version 2.5.2a). Alignments to the transcriptome output from STAR were used as input to Salmon (version 0.8.1) with position bias and GC bias corrections to obtain read counts and transcripts per million (TPM) at the transcript level. These transcript-level values were then aggregated to the gene-level using the tximport package in Bioconductor. Differential expression (DEx) analysis was performed on raw read counts per gene using DESeq2 with the various covariates. The *p* values were corrected using Benjamini-Hochberg. Genes were reported as statistically significant if the *p* value < 0.05.

### Gene ontology and GSEA

Gene ontology analysis and GSEA was performed using WebGestalt (http://www.webgestalt.org/)^22^ and MSigDB^23^.

### Reporting summary

Further information on research design is available in the Nature Research Reporting Summary linked to this article.

## Supplementary information

Supplementary Figures

Supplementary Tables

Reporting Summary

## Data Availability

The RNAseq data has been uploaded to the GEO database (GSE199468) [https://www.ncbi.nlm.nih.gov/gds/?term=(exosome%20gene%20expression%20technology%20to%20understand%20human%20health%20risk)%20AND%20gds_sra%5bfilter].
