# Supplementary material for: Exosome based analysis for Space Associated Neuro-Ocular Syndrome and health risks in space exploration
Source: NPJ Microgravity. 2022 Sep 14;8:40. doi: 10.1038/s41526-022-00225-4 (PMC9474550; doi:10.1038/s41526-022-00225-4)
Supplement: Supplementary file 1 — Supplementary Figures [file 41526_2022_225_MOESM1_ESM.pdf]

Supplementary Information for "Exosome based analysis for Space Associated Neuro-Ocular Syndrome and health risks in space exploration"

Sudipto K. Chakrabortty[1], Yevgenia L. Khodor[1], Robert R. Kitchen[1], Dulaney L. Miller[1], Kailey M. Babcock[1], Kyle S. Manning[1], Steven P. Lang[1], Vasisht Tadigotla[1], Wei Yu[1], Eric Bershad[2], Johan Skog[1] and Susana Zanello[3]

[1]Exosome Diagnostics, a Bio-Techne brand, Waltham, MA, USA

[2]Baylor College of medicine, Houston, TX, USA

[3]KBR, NASA Johnson Space Center, Houston, TX, USA

Supplementary Table 1: Gene sets significantly enriched in urine exosomes compared to plasma.

Supplementary Table 2: Gene sets significantly enriched in CSF exosomes when compared to plasma.

Supplementary Figure 1: Bar graph of the percentage of reads in each RNA-Seq library, by biofluid type, that were too short to be accurately mapped to the genome (≤ 30 nts). CSF samples have highest percentage of these reads.

Supplementary Figure 2: Dynamic range of detection of the total RNA-Seq libraries is estimated to be over 5 orders of magnitude, based on synthetic ERCC RNA spike-ins.

Supplementary Figure 3: Complete gene ontology analysis for high-ICP CSF samples compared to normal-ICP controls. Dashed on each graph is the significance cut-off point (Adjusted $p < 0.05$).

Supplementary Figure 4: A principal component analysis of post-Treatment urine samples compared to control urine samples yields no separation between sample types.

Supplementary Figure 5: Library preservation has little effect on gene expression of sequenced libraries.

(A) Biotype distributions for three individual urine RNA-Seq library samples sequenced immediately ("Fresh Library") or in one of three preservatives (DNAstable, GenTegra, and FTA Elute).
(B) Heatmap of sample correlations between fresh and preserved libraries shows that libraries cluster by sample source (Patient), not whether they were preserved.
(C) Principal component analysis of gene expression of three patient samples sequenced immediately ("Fresh") or using one of three preservatives. Libraries primarily cluster by sample source.

(D) Heatmap showing expression of significantly differentially expressed genes in all six libraries. Gene expression between fresh and preserved libraries for each patient is nearly identical.

Supplementary Figure 6: Sequencing metrics of matched preservative libraries before and after preservation.

(A) Pearson's correlation of ERCC spike-ins.
(B) Pearson's correlation of whole transcriptome expression level.
(C) Alignment statistics.
(D) Gene detection and diversity of exoRNA across biotypes.

Supplementary Figure 7: Distribution and overlap of exosomal RNA transcriptome across the 4 biofluids from the same individuals. Plasma, blood and CSF was collected at high ICP time point (t0) where urine was collected at low ICP time point (t1).

Supplementary Figure 8: Gene sets enrichment analysis (GSEA) of differentially expressed exoRNA between biofluids from matched individuals.

(A) Plasma to blood in high ICP conditions (cellular components).
(B) Plasma to blood in low ICP condition (cellular components).
(C) Plasma to urine in low ICP time point (biological processes).
(D) Plasma to CSF in high ICP time point (biological processes).

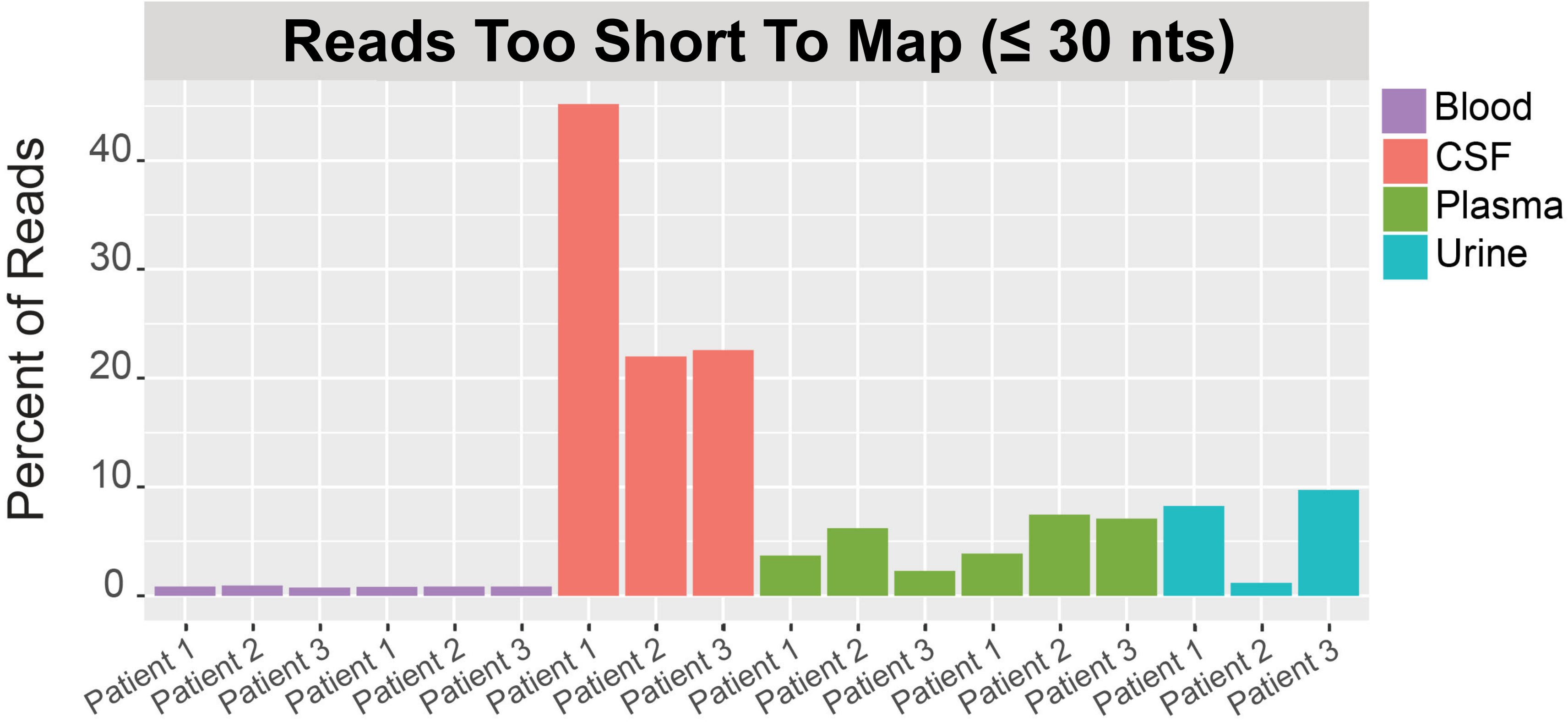

Supplementary Figure 1.

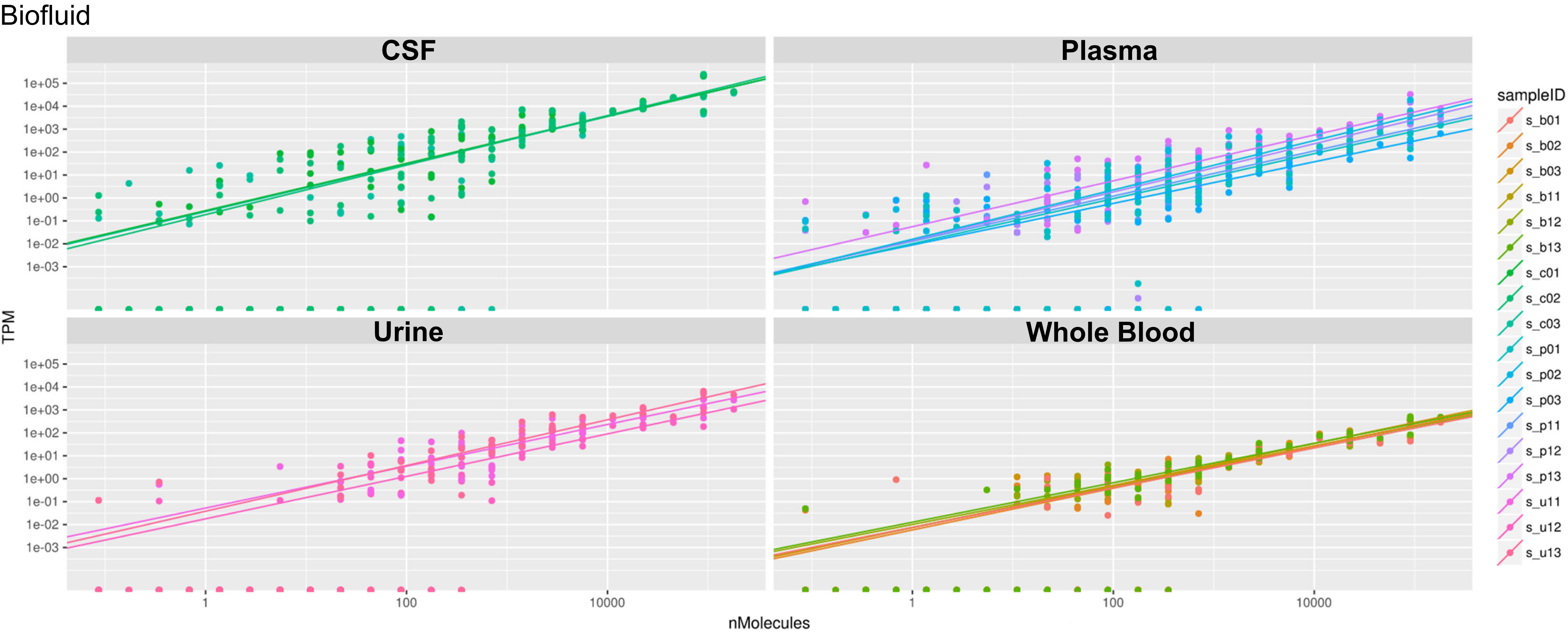

Supplementary Figure 2.

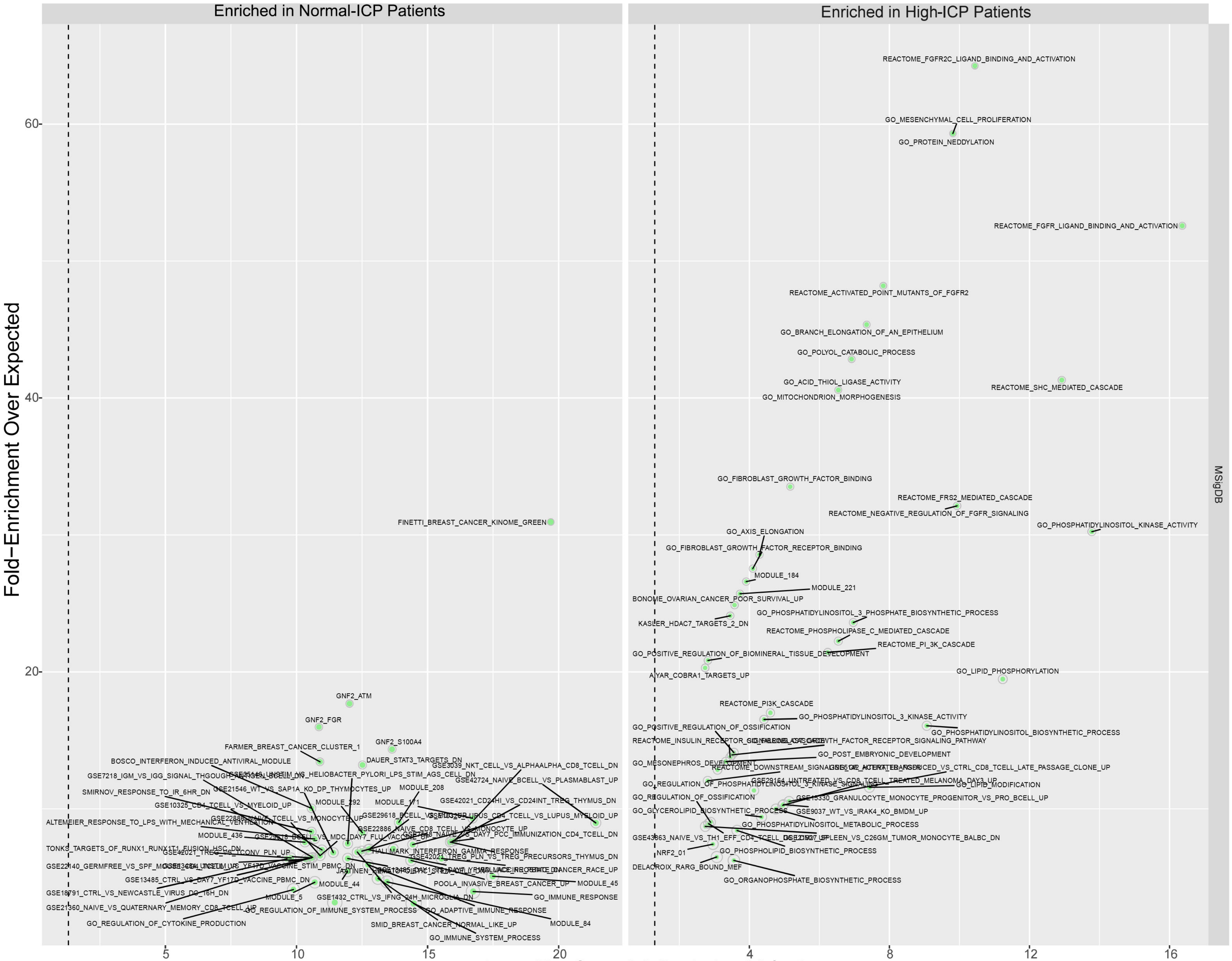

Supplementary Figure 3.

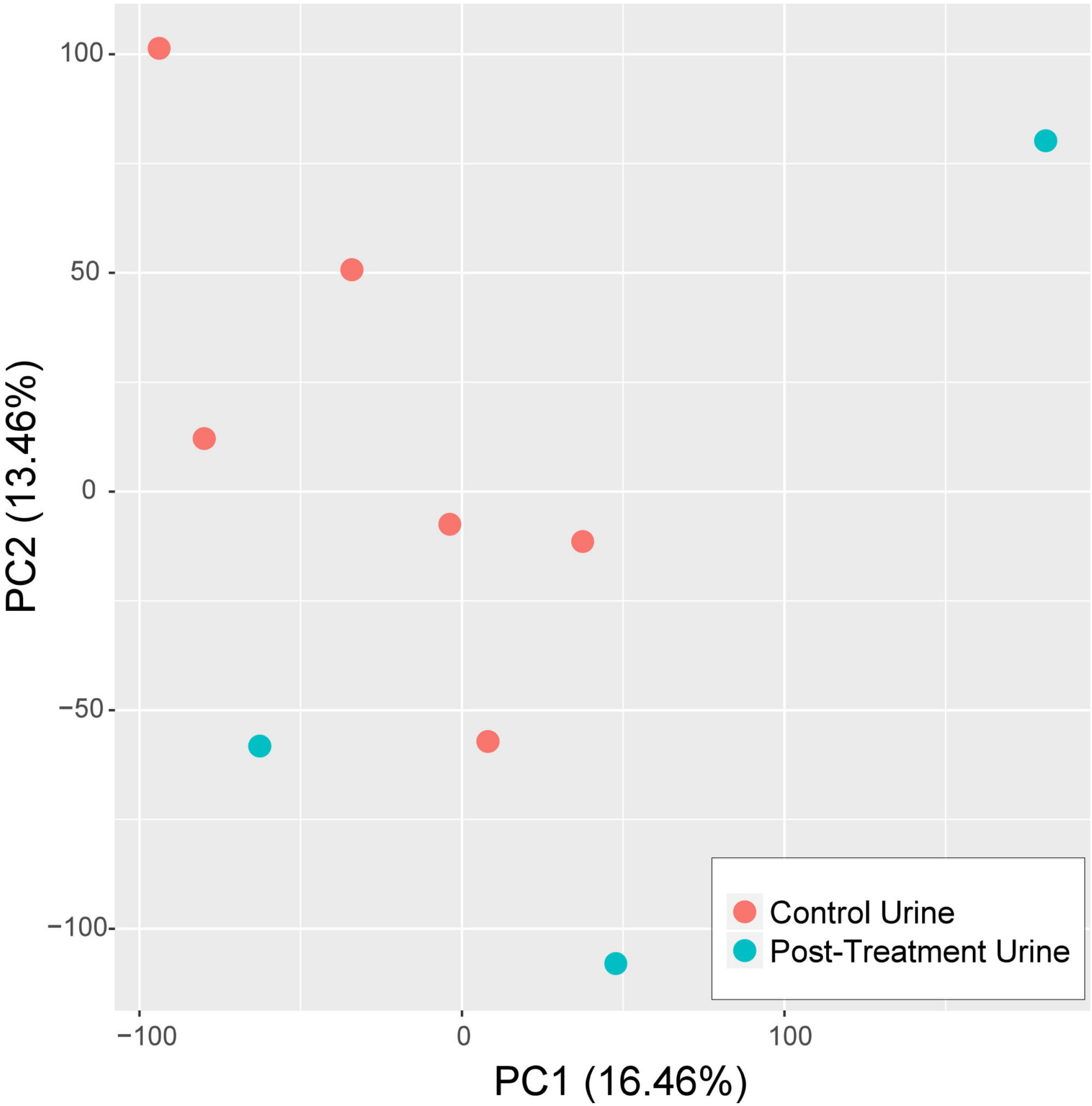

Supplementary Figure 4.

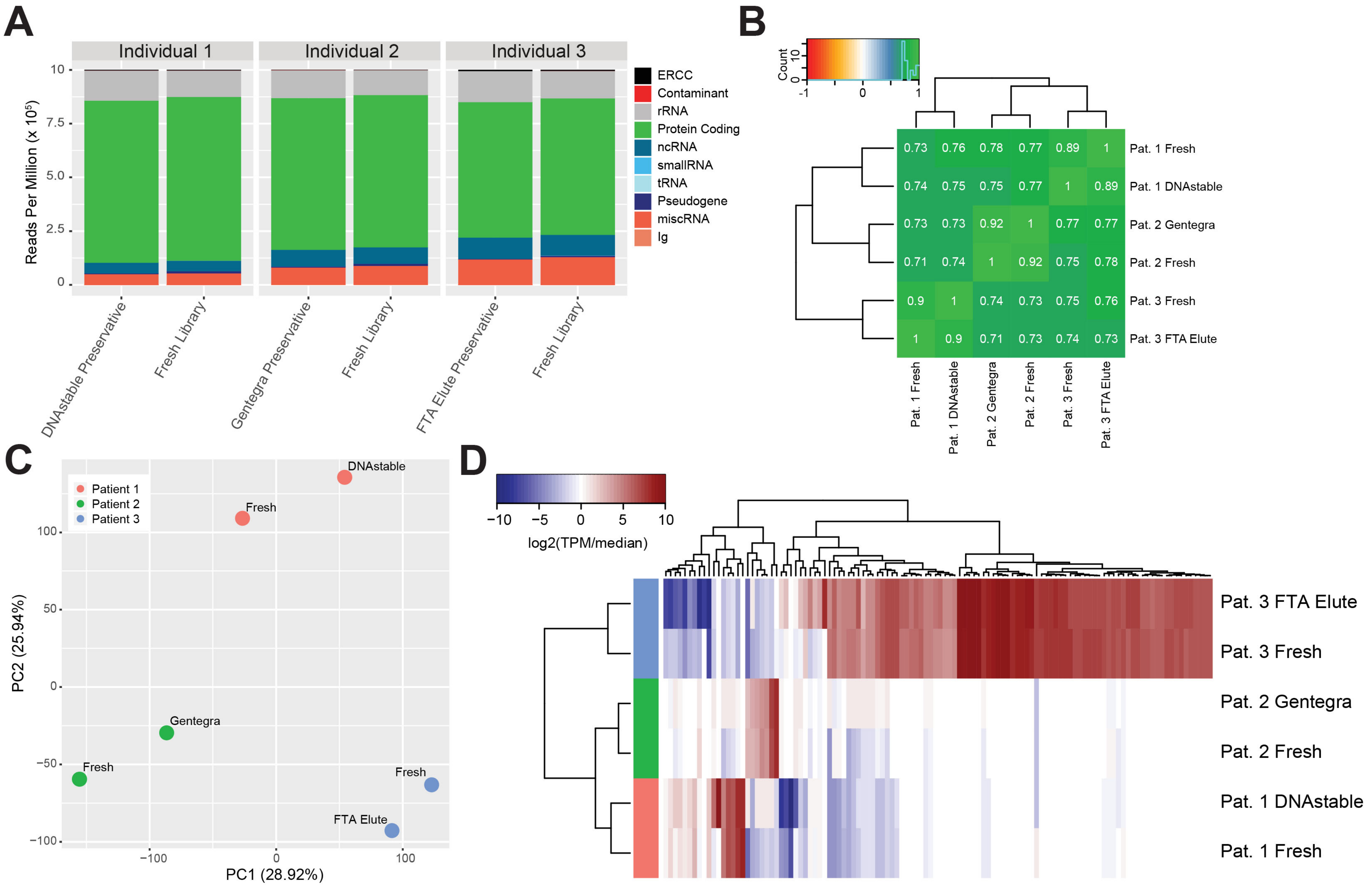

Supplementary Figure 5.

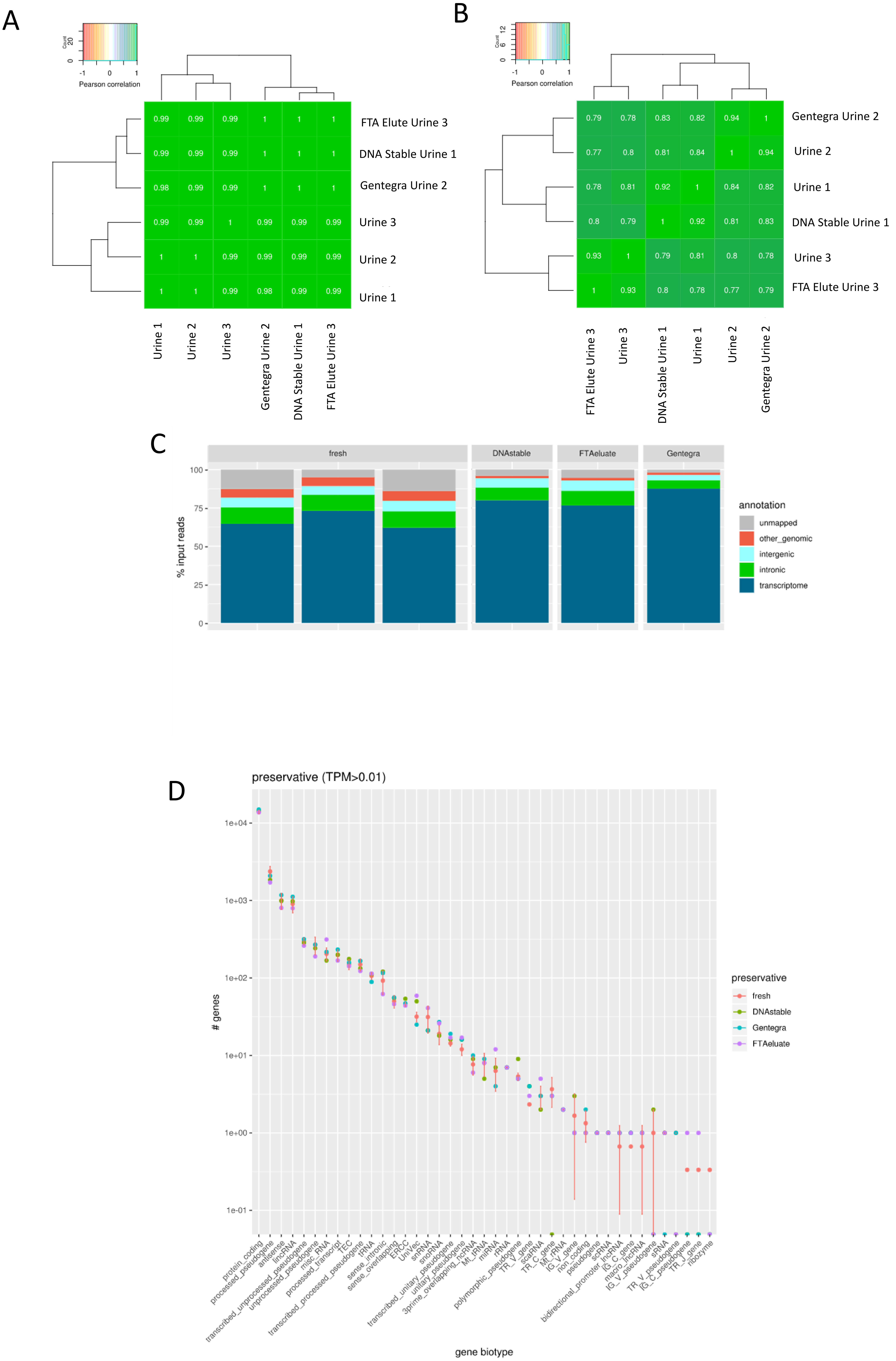

Supplementary Figure 6.

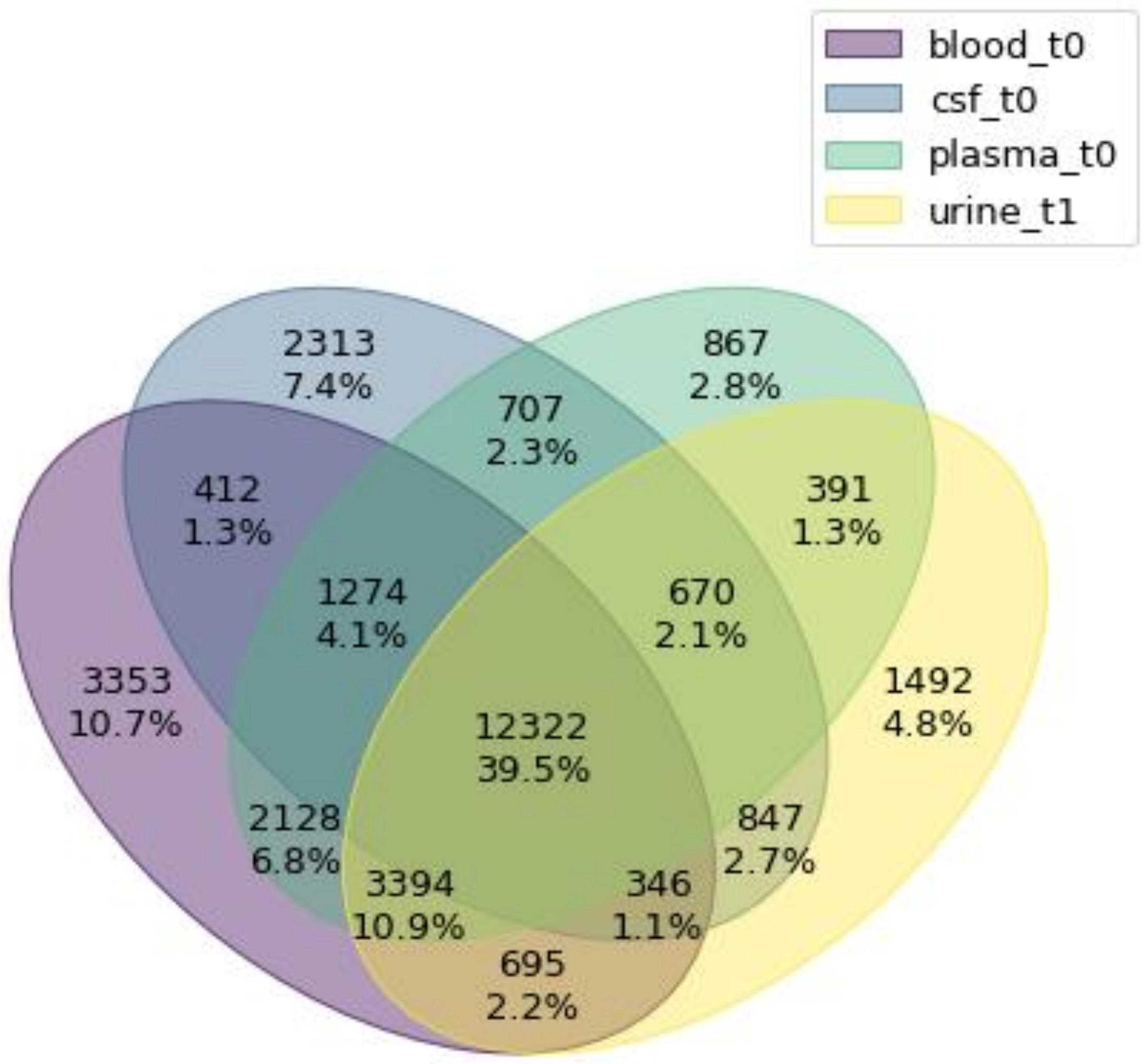

Supplementary Figure 7.

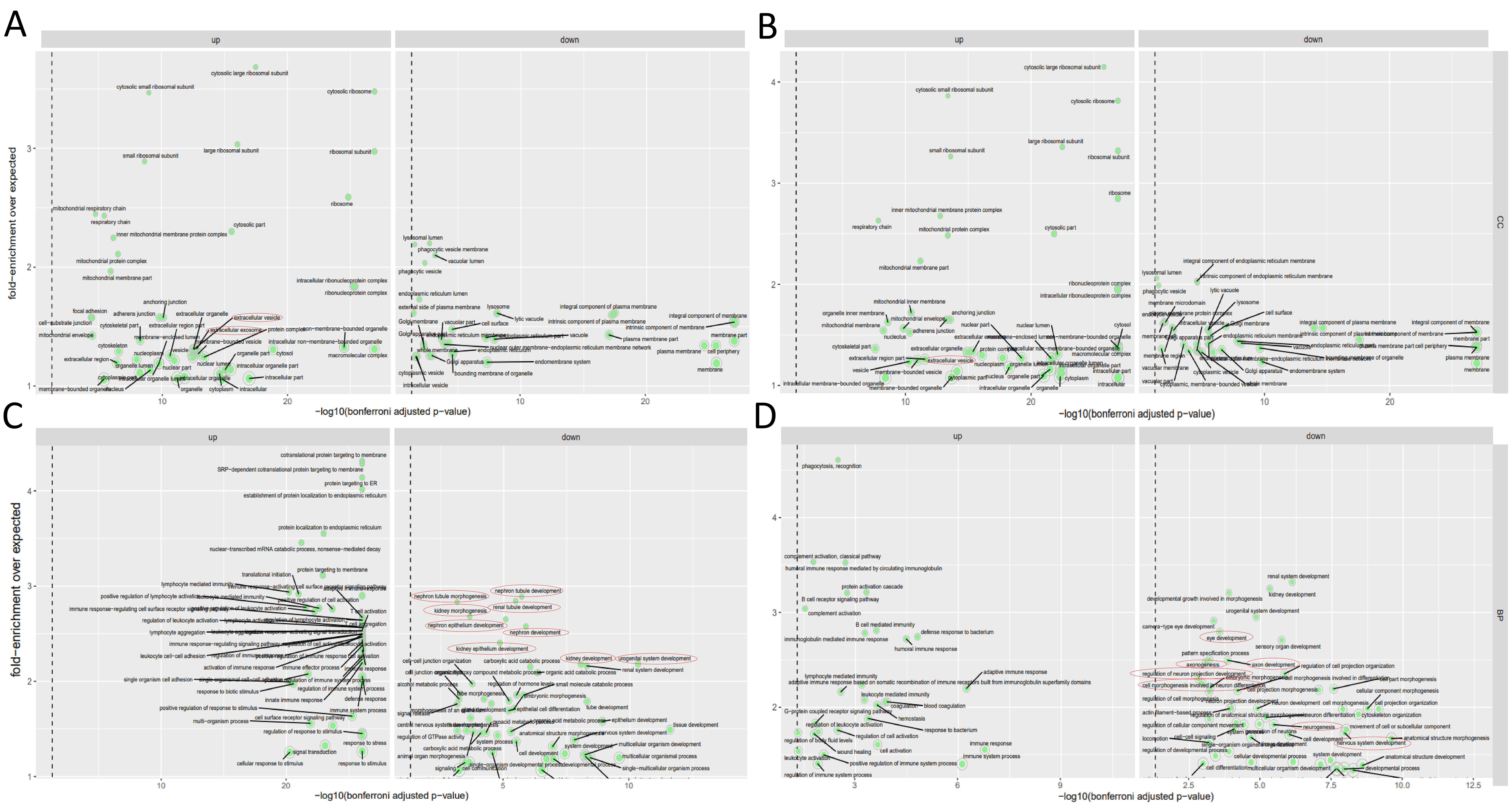

Supplementary Figure 8.
